# Research Progress on Dendritic Cells in Hepatocellular Carcinoma Immune Microenvironments

**DOI:** 10.3390/biom14091161

**Published:** 2024-09-16

**Authors:** Wenya Li, Guojie Chen, Hailin Peng, Qingfang Zhang, Dengyun Nie, Ting Guo, Yinxing Zhu, Yuhan Zhang, Mei Lin

**Affiliations:** 1 The Affiliated Taizhou People’s Hospital of Nanjing Medical University, Taizhou 225300, China; 2 Graduate School, Nanjing University of Chinese Medicine, Nanjing 210023, China; 3 Medical School, Nantong University, Nantong 226019, China; 4 The First School of Clinical Medicine Southern Medical University, Guangzhou 510515, China

**Keywords:** dendritic cells, hepatocellular carcinoma, tumor immunity

## Abstract

Dendritic cells (DCs) are antigen-presenting cells that play a crucial role in initiating immune responses by cross-presenting relevant antigens to initial T cells. The activation of DCs is a crucial step in inducing anti-tumor immunity. Upon recognition and uptake of tumor antigens, activated DCs present these antigens to naive T cells, thereby stimulating T cell-mediated immune responses and enhancing their ability to attack tumors. It is particularly noted that DCs are able to cross-present foreign antigens to major histocompatibility complex class I (MHC-I) molecules, prompting CD8^+^ T cells to proliferate and differentiate into cytotoxic T cells. In the malignant progression of hepatocellular carcinoma (HCC), the inactivation of DCs plays an important role, and the activation of DCs is particularly important in anti-HCC immunotherapy. In this review, we summarize the mechanisms of DCs activation in HCC, the involved regulatory factors and strategies to activate DCs in HCC immunotherapy. It provides a basis for the study of HCC immunotherapy through DCs activation.

## 1. Introduction

Hepatocellular carcinoma (HCC) is the most common malignant tumor of the liver, with an insidious onset and rapid progression of the disease. Most patients are diagnosed in the advanced stages of the disease and have limited therapeutic options; surgical resection, liver transplantation, local ablation, and other treatment modalities are no longer applicable, and the prognosis is usually poor. Targeted therapy, as a systemic therapy, prolongs the survival of patients, but its efficacy is still unsatisfactory due to the few therapeutic targets and the tumors’ resistance to targeted drugs [1]. In the search for effective therapeutic strategies, immunotherapy, which utilizes the immune system to kill tumor cells, has attracted extensive attention from researchers as a novel therapy for advanced HCC. The use of immune checkpoint inhibitors and dendritic cell (DC) vaccines has effectively improved patient survival and is a promising therapy [2]. DCs are integral components of the immune system, bridging innate and adaptive immunity by activating T cells, thereby generating anti-tumor immunity and effectively improving patient prognosis [3].

The activation of DCs refers to the process wherein these cells transition from a resting state to executing their functions under specific stimuli, which are crucial for initiating and coordinating anti-tumor immune responses. DCs activation enhances their antigen-presenting capabilities, and induces and augments T cell activation and proliferation, while also activating NK cells (natural killer cells), thereby bolstering the overall immune response against HCC. HCC patients often exist in an immunotolerant state; however, DCs activation can reverse this immunotolerance by stimulating immune cells and enhancing immune responses, thereby boosting the anti-tumor immune capabilities of HCC patients [4]. Here, the research advances on the mechanisms of DCs activation in the initiation and progression of HCC, the influencing factors, and activation strategies in immunotherapy are comprehensively reviewed, and the immunologic role of DCs activation in HCC is analyzed and summarized. This review aims to provide new insights for HCC immunotherapy research.

## 2. Dendritic Cell Subsets and Their Functions in Hepatocellular Carcinoma

DCs represent a heterogeneous group of immune cells that can be classified into different subsets based on their surface markers, tissue distribution, and other factors. The main subsets of DCs include plasmacytoid dendritic cells (pDCs) and conventional dendritic cells (cDCs) [5]. DCs, as antigen-presenting cells, play a crucial role in initiating T cell activation to exert anti-HCC immune effects. However, the unique anatomical location of the liver leads to immune tolerance towards antigens from the gut [6], while the presence of immune inhibitory molecules and cells in the tumor microenvironment inhibits the immune function of DCs. Therefore, the function of DCs is dynamically altered during the initiation and development of HCC.

pDCs are the primary producers of interferons (IFNs) and are mainly involved in antiviral and anti-tumor immune responses [7]. However, in HCC, the quantity and function of pDCs are often suppressed, resulting in insufficient immune surveillance and anti-tumor immune responses in the tumor microenvironment. Studies have reported the crucial role of pDCs in the postoperative recurrence of HCC, where the IFNα produced by pDCs drives the recruitment of myeloid-derived suppressor cells via the liver cell IRF1/CX3CL1 signaling pathway, creating an immunosuppressive environment that inhibits the tumor-killing activity of CD8^+^ T cells and promotes the postoperative recurrence of HCC [8].

cDCs are mainly divided into two major subsets: cDC1s and cDC2s [9]. cDC1s mainly express CD141^+^ (BDCA3^+)^, with BATF3 and IRF8 as their main transcription factors. In contrast, cDC2s predominantly express CD1c^+^ (BDCA1^+^), with IRF4 and ZEB2 as their main transcription factors [9]. cDC1s have potent antigen-processing and antigen-presentation capabilities, and are capable of degrading foreign antigens into small fragments and cross-presenting them to CD8^+^ T cells via major histocompatibility complex class I (MHC-1) molecules. cDC2s, on the other hand, present antigens to CD4^+^ T cells via MHC-II-like molecules. In addition to antigen presentation, DCs express costimulatory signals to promote T cell differentiation and induce specific T cell immune responses against tumor antigens [8,9]. Recently, it has been reported that cDC1s are also involved in the activation of CD4^+^ T cells [10]. Migratory CD103 cDC1s are able to transport cellular antigens from the periphery to the lymph nodes and have a strong ability to activate initial CD8^+^ T cells in vitro, whereas CD11b cDC2s have a limited ability to process antigens, which leads to the conclusion that cDC1s are the major subset of cDC1s that exert antitumor immunity in vitro [11].

However, tumors tend to shape an immunosuppressive tumor microenvironment, impairing or inhibiting the function of DCs. For example, the HCC-related antigen alpha-fetoprotein (AFP) can impair DC function, promoting malignant tumor progression in AFP-positive HCC patients [12]. Thus, DCs play an important role in anti-tumor immunity and maintaining immune homeostasis, and enhancing the tumor-killing ability of DCs is crucial for cancer therapy.

## 3. Activation of Dendritic Cells in Hepatocellular Carcinoma

The activation of DCs represents a crucial initial step in triggering immune responses; they recognize pathogen-associated molecular patterns (PAMPs) or damage-associated molecular patterns (DAMPs) through cell surface pattern recognition receptors (PRRs), leading to the activation of signaling pathways mediated by adaptor proteins such as TRIF and MyD88, thereby inducing phenotypic and functional changes in DCs [11,13,14,15].

PAMPs are evolutionarily conserved components (such as lipopolysaccharides, and bacterial and viral nucleic acid components) found in pathogenic microorganisms, while DAMPs are endogenous molecules released from damaged or dying cells, such as heat shock proteins (HSPs) and high mobility group protein 1 (HMGB1). Toll-like receptors (TLRs), a crucial class of pattern recognition receptors, perceive both PAMPs and DAMPs [15,16,17]. TLR4, the first characterized TLR, activates DCs by recognizing lipopolysaccharide (LPS) [18,19]. LPS has become a classic stimulus molecule for activating bone marrow-derived dendritic cells (BMDCs) [20]. The signaling pathways involved in DC activation mainly include the p38MAPK, ERK, JNK, PI3K/AKT, and NF-κB signaling pathways [21,22,23], among which, the PI3K/Akt pathway is crucial for the survival of DCs [24]. HMGB1 released by cancer cells after chemoradiotherapy can be recognized by TLR4 and activate DCs [25]. However, studies have reported that tumor-derived DNA, after recognition by DCs, is sensed by cGAS-STING and activates DCs to secrete the cytokine IFN-β [26]. The involvement of cGAS-STING in DC activation and secretion of IFN-β has also been observed In HCC tumor models [27,28]. Surprisingly, a recent study on multifunctional nanoparticles enhancing HCC immunity has supported the role of the cGAS-STING signaling pathway. Metal–organic framework 801 (MOF-801) can act as a STING agonist and is recognized by TLR4, activating the cGAS-STING/NF-κB signaling pathway, thereby promoting DC maturation and IL-6 secretion [29].

Immature DCs can effectively capture and take up antigens in non-lymphoid tissues, during which, the expression of the chemokine receptor CCR7 on the cell surface increases, guiding DCs to migrate to lymphoid organs such as lymph nodes. In lymph nodes, these DCs undergo a process of maturation or activation [30]. To be more precise, MHC molecules (whose expression is increased) present antigenic peptides to naive T cells, which is the first signal for T cell activation. Activated DCs also express various co-stimulatory molecules such as CD80 and CD86, which bind to CD28 on T cells, providing the second signal for T cell activation. Ultimately, activated DCs secrete large amounts of cytokines such as IL-12, promoting T cell differentiation and providing the third signal for T cell activation [31]. These three signals are indispensable for T cell activation, and the expression of the associated molecules during signal transduction is a key feature of DC activation. The expression levels of CD40, CD54, CD70, CD80, CD83, CD86, CCR7, and MHC molecules are commonly used to assess the activation state of DCs in HCC immunotherapy [21,32,33,34,35,36,37]. The activation of DCs promotes their antigen-presenting ability, which is crucial for T cell activation, induces the differentiation of T helper 1 (Th1) cells, recruits specific lymphocytes in HCC, allows them to exert their immune function, and inhibits the malignant progression of HCC [23,34,38,39,40,41]. Activated DCs secrete the pro-inflammatory factor IL-12, which promotes the activation of NK cells and participates in the anti-HCC process [27].

## 4. Regulation of Dendritic Cells Activation in Hepatocellular Carcinoma

**Figure 1:**
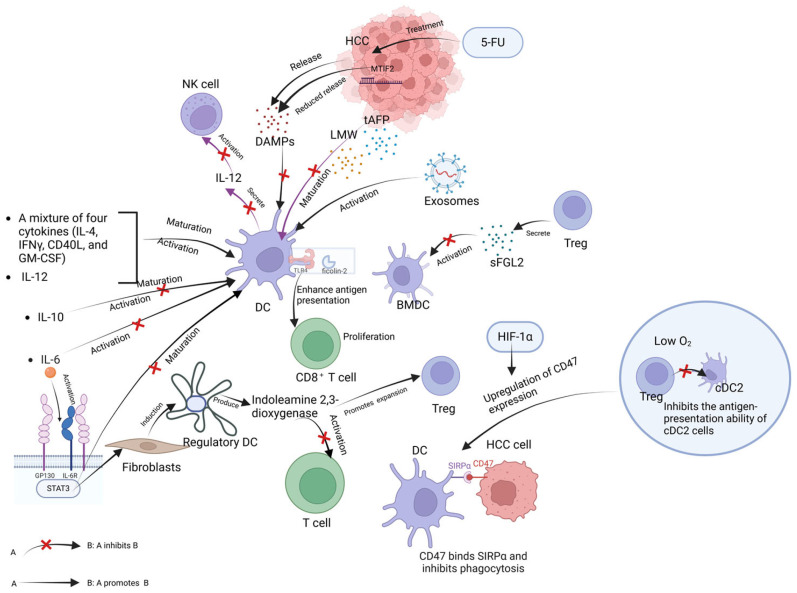
Schematic representation of factors affecting DC activation in the immune microenvironment of hepatocellular carcinoma. Created with BioRender.com.

The environment in which tumors reside is known as the tumor microenvironment (TME), which comprises HCC cells, immune components surrounding the tumor, and non-immune stromal components, which all interact to mediate the growth of the HCC and the balance of anti-tumor immune responses [42,43]. DCs, as antigen-presenting cells (APCs) involved in cellular immune responses, are subject to complex and dynamic regulation by the HCC tumor microenvironment. In addition to HCC cells themselves, both immune and non-immune stromal components of the tumor can produce various cytokines that participate in the tumor immune response. Cytokines, tumor-associated antigens, lectins, exosomes, and regulatory T cells (Tregs) are all part of the HCC tumor microenvironment and participate in the regulation of DC activation. Figure 1 shows the influencing factors of DC activation in the immune microenvironment of HCC and illustrates the role of components of the immune microenvironment in relation to DCs.

### 4.1. Cytokines

Cytokines, a class of secretory proteins, participate in regulating immune responses, tumor growth, and inflammation processes [44,45,46,47]. In HCC immunity, various cytokines are involved in regulating the immune response, with some serving as key regulatory factors that modulate DC activation [48,49,50,51]. IL-10, an immunosuppressive cytokine, has been shown in multiple studies to downregulate DC surface co-stimulatory molecules and MHC class II antigens. In vitro experiments with HCC have demonstrated that the release of IL-10 inhibits DC maturation and activation [52]. IL-6 is produced by a variety of cell types, including cancer cells, fibroblasts, immune cells, and endothelial cells [53], and its expression is increased in a variety of cancers [54], especially in the serum of liver cancer patients [55], and it is a cytokine that is closely associated with poor prognosis in HCC and is involved in HCC progression [56,57]. Several studies have shown that IL-6 inhibits DC differentiation, resulting in reduced numbers and impaired function of mature DCs, which is closely related to IL-6-mediated STAT3 activation [58,59,60]. IL-6-mediated STAT3 activation is followed by decreased expression of DC maturation markers and the subsequent inhibition of effector T cell activation [59,61]. Additionally, the induction of regulatory DCs generation by hepatic carcinoma-associated fibroblasts is also associated with IL-6-mediated STAT3 activation; regulatory DCs produce indoleamine 2,3-dioxygenase, which inhibits T cell function and promotes Treg expansion, exerting immunosuppressive effects [53]. IL-12, as a cytokine that activates TH1 immune responses, participates in DC activation. The induction of IL-12 expression by DCs significantly increases the proportion of activated DCs, promotes the expression of co-stimulatory molecules, and induces potent anti-HCC responses [62]. A study on HCC immunotherapy reported that a mixture of four cytokines (IL-4, IFNγ, CD40L, and GM-CSF) co-cultured with immature DCs isolated from the spleen enhanced the antigen-presenting ability of DCs and increased the expression of maturation markers, indicating the powerful effect of these cytokines in activating DCs [34].

### 4.2. Tumor-Associated Antigens

Alpha-fetoprotein (AFP) is a tumor-associated antigen in HCC [40]. Previous studies have found that it inhibits the maturation of DCs. Subsequent research indicated that HCC tumor-derived AFP (tAFP) synergizes with low-molecular-weight (LMW) molecules in impairing DC differentiation and function, with LMW molecules being necessary for tAFP to reduce the expression of DC maturation markers [63,64]. Additionally, AFP inhibits the production of IL-12 by DCs [40], thereby impairing the activation of NK cells. Due to its inhibition of DC maturation, AFP may also induce the killing of immature DCs by NK cells [65]. Mitochondrial Translational Initiation Factor 2 (MTIF2) is highly expressed in HCC and serves as a central tumor immune infiltration gene, inhibiting DC maturation by reducing the release of DAMPs after 5-fluorouracil (5-FU) treatment [66].

### 4.3. Complement Lectins

Ficolin-2, a complement lectin, is lowly expressed in the serum of cancer patients. It plays a significant role in maintaining immune balance by binding to pathogens to activate the complement system, participating in inflammation regulation, and activating immune cells [67,68]. Ding et al. demonstrated that ficolin-2 activates DCs by binding to TLR4 on DCs, thereby enhancing the proliferation of CD8^+^ T cells. The activation of immune cells by ficolin-2 contributes to inhibiting malignant liver cancer progression, providing strong support for ficolin-2 as an immunotherapeutic agent [69].

### 4.4. Exosomes

Exosomes are double-layered membrane vesicles secreted by cells, which contain various lipids, proteins, and nucleic acids and play crucial roles in physiological and pathological processes. Shi et al. found that the level of serum exosomes in HCC patients was significantly higher than in normal individuals, and exosomes could promote DC activation, increase the secretion of IL-2 and IL-12, and mediate T cell responses to exert anti-tumor effects [70].

### 4.5. Tregs

Tregs are a type of immune cells and are primarily responsible for regulating and suppressing immune responses to maintain immune system balance and self-tolerance [71,72]. They typically inhibit immune responses against tumor antigens, thereby promoting tumor growth [73]. Some studies have shown that Tregs are overexpressed in HCC patients and are often associated with poor prognosis [74,75,76] and mediating HCC immune escape [73]. Soluble fibrinogen-like protein 2 (sFGL2) secreted by Tregs in HCC downregulates the expression of DC-related inflammatory markers on BMDCs by suppressing the phosphorylation of AKT and p38. Additionally, the increase in Tregs promotes the secretion of IL-35, resulting in the suppression of immune cell numbers, including DCs, which may contribute to the poor efficacy of HCC immunotherapy [23].

### 4.6. Hypoxia

Hypoxia is a significant characteristic of the tumor microenvironment due to the rapid growth of tumor cells, which often leads to oxygen deficiency in tumor tissues [77]. Hypoxia affects DC activation through multiple mechanisms. TME hypoxia can induce the upregulation of CD47 expression in HCC cells, mediated by hypoxia-inducible factor 1α (HIF-1α). CD47 can interact with its receptor signal regulatory protein α (SIRPα) to inhibit the phagocytic activity of DCs and promote tumor immune escape [27,78]. Furthermore, in highly hypoxic regions of liver cancer, Tregs and cDC2s are significantly enriched, with Tregs mediating the downregulation of HLA-DR expression on cDC2s, inhibiting the antigen presentation ability of cDC2s [79].

## 5. DC Activation Strategies in HCC Immunotherapy

**Figure 2:**
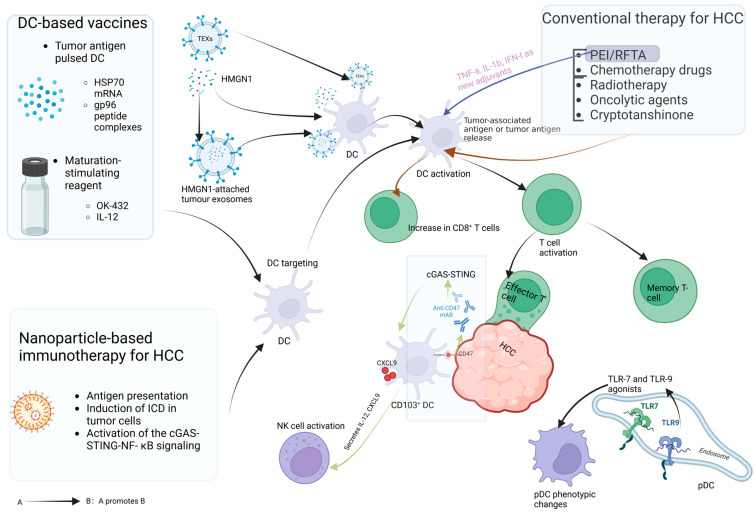
Dendritic cells in HCC immunotherapy. Created with BioRender.com.

Immunotherapy based on DCs holds great promise in the treatment of HCC. The activation of DCs facilitates the induction of immune responses against HCC cells, thereby reversing the immunosuppressive tumor microenvironment, which is a crucial step in immunotherapy. Currently, various strategies in HCC immunotherapy, including targeted therapy, nanoparticle delivery, and vaccines, are employed to activate DCs for better tumor responses. The specific activation mechanisms and effects of these strategies are outlined in Table 1. Meanwhile, Figure 2 demonstrates the strategies to activate DCs in HCC immunotherapy in a visual way.

### 5.1. DC Vaccines

DC-based vaccines in HCC immunotherapy leverage their antigen-presenting properties to deliver HCC-associated antigens to the immune system, stimulating effective anti-tumor immunity. To achieve this, several strategies have been developed to load tumor antigens onto DCs. To overcome the limitation that peptides of tumor-specific antigens require specific HLA molecules for presentation [80], several strategies have been developed to load tumor antigens onto DCs to present multiple tumor antigens. These strategies include loading DCs with tumor lysates/tumor cell RNA, transfecting DCs with plasmid DNA/viral vector DNA/mRNA encoding known antigen genes, and fusion of DCs with tumor cells [81,82,83,84,85]. However, these strategies are often combined with other methods to stimulate DCs, such as adjuvants and drugs, to better activate DCs and exert anti-tumor effects. HSPs, stress-induced molecular chaperones, can act as natural adjuvants to efficiently present peptide partners to APCs via specific receptors. Moreover, HSPs are able to effectively stimulate the secretion of cytokines and chemokines, inducing DCs maturation. A completed phase I clinical trial assessed the safety and feasibility of HSP70 mRNA-transfected DC therapy for unresectable or recurrent HCC treatment [86]. Subsequent phase I/II randomized controlled trials validated its safety and efficacy as an adjuvant therapy after curative resection for HCC. The vaccine was safe, well-tolerated, and resulted in significantly longer overall survival in the DC group compared to the control group in the HCC subgroup expressing HSP70 [87]. Additionally, Wang et al. utilized human-derived HSP-gp96 peptide complexes prepared from HCC SMMC-7721 cells as antigens for pulsing DCs, promoting DCs activation, stimulating autologous T cell proliferation, and inducing specific T cell generation [81]. Similarly, adding microbial HSP70 peptide epitopes 407–426 and an anticancer agent (OK-432) to pulsed DCs effectively enhanced DCs activity and the efficacy of DC vaccines [88]. However, the immune microenvironment is a dynamic and holistic system. Activating DCs may lead to changes in other cells. Annabelle Vogt et al. [62] pointed out DC transduction with IL-12-encoded adenovirus was accompanied by an increase in immunosuppressive cells while activating Th1 immunity. The increase in immunosuppressive cells may be to prevent excessive inflammation and autoimmunity. This can be improved by combination therapy with drugs regulating other immunosuppressive cells. Currently, antigen-loaded DC vaccines have entered clinical trial stages and are being combined with other immunotherapies for HCC treatment. The first stage of a phase II trial (NCT03067493) assessed the safety of an antigen-loaded DC vaccine combined with T cell immunotherapy. Among the 10 patients, no grade 3 or higher treatment-related adverse events were reported, 70% of patients developed antigen-specific T cell responses, and 71.4% of patients remained recurrence-free within two years after curative treatment, demonstrating the safety and feasibility of this therapy [89]. PD-L1 is a ligand on tumor cells that binds to the PD-1 receptor on T cells and can inhibit T cell proliferation and activation and participate in the immune escape of tumors. Therefore, blockade of the PD-1 pathway by PD-1/PDL-1 inhibitors can restore the ability of T cells to kill tumor cells [90]. Clinical trials conducted with PD-1/PDL-1 inhibitors have shown that they are safe and effective for the treatment of HCC [91]. Currently, they are commonly used as an adjuvant therapy for HCC. A recent study showed that a tumor lysate-pulsed DC vaccine combined with a PD-L1 inhibitor had a stronger anti-tumor T-cell response and better prognosis compared to monotherapy [92]. Therefore, PD-1/PDL-1 inhibitors combined with DC vaccines for the treatment of HCC hold great promise.

### 5.2. Exosome-Related Immunotherapy

Tumor-secreted exosomes can serve as delivery tools to transport various tumor antigens to DCs, triggering DCs activation and exerting potent tumor-specific killing effects. A study on exosome stimulation of HCC immunity revealed that tumor-derived exosome (TEX)-pulsed DCs exhibited a stronger immune response than cell lysate-pulsed DCs, significantly inhibiting tumor growth and reshaping the immune microenvironment (increased levels of immune stimulatory factors, decreased levels of inhibitory factors). Surprisingly, HCC TEXs not only cross-protect HCC cells from mice and humans but also exert effects on pancreatic cancer cells, demonstrating their broad potential as antigen carriers [93]. Additionally, the addition of high-mobility group nucleosome-binding protein 1 (HMGN1) as an adjuvant promotes the immunostimulatory effect of TEXs on DCs, while HMGN1 itself can promote the recruitment and activation of DCs [94]. TEXs painted with the HMGN1 functional domain and DC-derived exosome vaccines painted with the HMGN1 functional domain both promote DCs activation, especially the latter, which can promote the activation of endogenous DCs in HCC, thereby presenting antigens and inducing tumor-specific T cell responses [37,95]. In summary, exosome-related immunotherapy is a promising immunotherapy strategy.

### 5.3. Targeted Therapy

pDCs highly express TLR7 and TLR9. TLR7 and TLR9 agonists can stimulate pDCs to sense infections and induce the phenotypic maturation of pDCs and expression of IFN-α. However, consideration should be given to the immune limitations brought about by TLR stimulation of pDCs. Therefore, it is crucial to develop activators that can avoid excessive activation of pDCs while maintaining their immune function for the treatment of HCV (hepatitis C virus)-related HCC [36]. In addition to Toll-like receptor agonists, the use of receptor blockers is also a way to activate DCs.

CD47 is a protein that is overexpressed on multiple tumor cells, inhibiting the phagocytic function of DCs by binding to SIRPα [96]. Therefore, in HCC immunotherapy, the use of CD47-blocking agents can disrupt its binding, restore the phagocytic function of DCs, enhance their antigen processing and presentation capabilities, thereby activating DCs and enhancing the immune responses [27].

### 5.4. Nanocarrier-Based Immunotherapy for HCC

Chemotherapy drugs, radiotherapy, local ablation, oncolytic agents, and certain compounds can be categorized as immunotherapy, but their mechanisms primarily involve damaging HCC cells, leading to the release of endogenous adjuvants and tumor antigens, indirectly activating DCs and inducing anti-tumor immune responses [32,33,97,98,99]. After the local ablation of the HCC, TNF-α and IL-1β in the patient’s serum can act as new adjuvants to promote the maturation of myeloid dendritic cells [97]. Therefore, combining DC-related immunotherapy with local ablation may reverse tumor immunosuppression, potentially offering an effective treatment strategy for HCC.

Nanocarriers are nanoscale materials capable of carrying and releasing biologically active substances such as drugs, antigens, and genes. By modulating their structure, composition, and surface properties, nanocarriers can exert protection, control release, and target delivery of drugs or active substances, making them more easily recognized and engulfed by APCs, thus playing an important role in HCC immunotherapy [100,101,102]. Previously, RNA-pulsed DC vaccine preparation required long cycles and high costs. But the emergence of lipid nanoparticles loaded with HCC RNA greatly shortened the vaccine production time while protecting RNA stability and delivering RNA to DCs, inducing DCs activation and anti-tumor activity both in vivo and in vitro [103].

The delivery of antigens and Toll-like receptor agonists by nanoparticles can activate the maturation of DCs, promote potent T cell responses, and inhibit the malignant progression of HCC [104,105]. After radiotherapy and microwave ablation, tumors release “danger signals”. Mannose-derived carbon dots and mesoporous silica nanoparticles can capture these signals, enhance antigen presentation by DCs, and increase immune cell infiltration [106,107]. A recent study showed that a nanoparticle drug that captures tumor-associated antigens (TAAs) released after thermal ablation delivers antigens and drugs promoting DC maturation to tumor-infiltrating dendritic cells (TIDCs), stimulating TIDC activation and maturation, thereby presenting antigens to T cells to activate anti-tumor immunity [108]. Creating an immune-stimulating microenvironment is crucial for DCs activation. A nanovaccine carrying cGAMP and adsorbed TAAs was designed to trigger persistent immune responses, assisting radiofrequency ablation therapy [109]. In addition to loading antigens, nanocarriers deliver drugs that induce immunogenic cell death (ICD) of HCC cells [110], activating DCs by releasing DAMPs and TAAs, and reversing the immunosuppressive tumor microenvironment to promote an immune-supportive microenvironment [4,111,112]. Furthermore, nanoparticles amplified by autophagy have been reported to promote tumor immunity. Exploiting the sensitivity of tumor tissue to autophagy after radiofrequency ablation breaks the protective effect of low levels of autophagy on tumor cells, relieving their immune suppression and inducing ICD of tumor cells, thereby promoting DCs maturation [113]. Surprisingly, metal–organic framework 801 (MOF-801) not only serves as a delivery vehicle but it also, when combined with other drugs as a STING agonist, activates the NF-κB signaling pathway to reprogram tumor-associated macrophages and accelerate DCs maturation [29]. In summary, nanocarriers have a short preparation time, good targeting, and can load multiple active substances to activate DCs, induce potent and durable anti-tumor immunity, and reshape the tumor microenvironment, showing broad prospects in the treatment of HCC. Notably, the DCs in HCC immunotherapies that we discussed above were mainly obtained by adding GM-CSF and IL-4 to PBMCs or mouse bone marrow cells in culture, which are also known as MoDCs (monocyte-derived dendritic cells) or BMDCs [114]. They are phenotypically and functionally different from naturally occurring cDCs and are similar only under specific conditions. A growing number of studies have shown that in vitro-obtained DCs have lower activity and function compared to naturally occurring DCs (nDCs), and moDCs do not cross-present as well as nDCs [115]. pDCs and cDCs are considered nDCs, and among the several DC subsets currently available, cDC1 has a greater ability to cross-present antigenic antigens and a strong ability to activate CD8^+^ T cells in vitro. In addition to this, pDCs are effective in cross-presenting exogenous antibodies to CD8^+^ T cells despite their low uptake of antigens, and myeloid DCs are able to induce a Th1 response through TLR-mediated stimulation to produce IL-12. Similarly, pDC-derived type I IFNs can act as Th1-inducing cytokines and participate in Th1 differentiation. In conclusion, pDCs show strong potential in activating anti-tumor immunity [116].

**Table 1:** Activation strategies for dendritic cells and their effects in hepatocellular carcinoma immunotherapy.

DC Therapy	DC Activation Method	Principle	Effects
DC vaccines	Dendritic cells pulsed with gp96-peptide complexes (in vitro) [81]	Antigenic stimulation	Increased expression of MHC class II, CD80, CD86, CD40, and CD83; generation of specific CTLs
	mHSP70 407-426 and OK-432 tumor cell lysate-pulsed DCs (in vitro and in vivo) [88]	OK-432 promotes DC maturation; mHSP70 407-426 can improve DC maturation through enhancing the interaction between CD40 and CD40L	Activation of DCs; increased production of Th1-type cytokines; induction of lymphocyte proliferation and high levels of CTL production; effective inhibition of tumor growth
	Tumor-lysate pulsed DCs were transduced with IL-12-encoding adenoviruses (in vitro and in vivo) [62]	IL-12 promotes the expression of CD83 and co-stimulatory molecules	Induced robust Th1 immune responses and tumor cell apoptosis
Exosome-related immunotherapy	TEX-pulsed DCs (in vitro and in vivo) [93]	Antigenic stimulation	Increased number of T lymphocytes; significant inhibition of tumor growth; increased levels of IFN-γ; decreased levels of IL-10 and TGF-β
	HMGN1-attached tumor exosomes (in vitro and in vivo) [95]	HMGN1 promotes the maturation and activation of DCs	Promoted DCs activation, generated memory T cells and enhanced anti-tumor immunity
	A DEX vaccine loaded with P47-P, AFP212-A2, and N1ND-N (in vivo) [37]	HMGN 1 promotes DC recruitment and activation	DC uptake and cross-presentation of tumor neoantigens triggered robust tumor-specific immune responses
Receptor agonists	TLR7 and TLR9 agonists (in vitro) [36]	Stimulation of pDCs to sense infections induces pDCs phenotypic changes	IFN- α, CD86, CD40, and HLA-DR were upregulated
Receptor blockers	CD47 blocking agents (in vitro and in vivo) [27]	Blocking the binding of CD47 to signal-regulated protein α restores phagocytosis in dendritic cells and activates them	Activation of the cGAS-STING pathway promoted CD103^+^ DCs secretion of CXCL9 and IL-12 and activation of NK cells
Local ablation	PEI/RFTA (in vitro) [97]	Promotes a pro-inflammatory environment in the serum after PEI/RFTA treatment	Increased serum levels of TNF-a and IL-1β; promoted transient activation of MDCs in peripheral blood and stimulation of CD4^+^ T cells
Chemotherapy drugs	Hyperbaric oxygen and teniposide combination therapy (in vitro and in vivo) [99]	Hyperbaric oxygen significantly enhances teniposide-induced cGAS-STING-dependent type I tumor interferon and NF-κB signal transduction	Activation of DCs and tumor-infiltrating CTLs enhanced sensitivity to anti-PD-1 immunotherapy
Radiotherapy	Irradiation (in vivo) [32,33]	Irradiation leads to the release of tumor-associated antigens	Activation of DCs and T cells; the expression of CD86 and MHC class II increased; and the number of CD11c^+^ DCs and CD8^+^T cells increased
Oncolytic agents	WNV live attenuated vaccine (in vitro and in vivo) [117]	Tumor antigen release is induced by oncolytic viruses	Activation of DCs and CD8 ^+^ T cells; inhibition of tumor proliferation
Compounds	Cryptotanshinone (in vitro and in vivo) [98]	Increases the supply of tumor antigens available to the immune system	Inhibition of HCC cell proliferation; activation of DCs and macrophages; increased CD8T cell infiltration
Nanoparticle-based immunotherapy for HCC	Lipid nanoparticles loaded with HCC RNA (in vitro and in vivo) [103]	Antigen presentation	Promoted DC maturation and induced specific CTL production; effectively inhibited growth of HCC in mice
	DOX/ ICG-loaded BHM nanoparticles (in vitro and in vivo) [111]	Induces ICD in tumor cells	Promoted DC activation and CD8^+^T cell and CD4^+^T lymphocyte infiltration; improved immunosuppressive TME
	SSZ-loaded CH-OD(CH-OD-SSZ) hydrogel (in vitro and in vivo) [4]	Induces ICD in tumor cells and increases expression of DAMPs to induce DC maturation in a co-culture model	Increased expression of CD80 and CD86; Repolarization of macrophages to M1-like phenotype; TIME remodeling; enhanced anti-tumor immunity
	H-ferritin nanocages loaded with doxorubicin (in vivo) [112]	Induces ICD in tumor cells	Promoted the activation and maturation of DCs to exert a powerful tumor-killing effect
	A virus-like silicon vaccine with a unique spike topological structure (in vitro and in vivo) [104]	Co-delivery of neoantigen and TLR9 agonist to DCs	Activated DCs that promote robust CD8^+^ T cells and central memory T cells responses to inhibit orthotopic HCC tumor growth
	MOF-CpG-DMXAA (in vivo) [29]	Activates the cGAS-STING–NF-κB signaling pathway	Reprogrammed TAMs; promoted the maturation of DCs; increased infiltration of CD4^+^ and CD8^+^ T cells; decreased expression of Tregs; and generation of potent tumor-killing immunity

DC: dendritic cell; MHC class II: major histocompatibility complex class II; CTLs: cytotoxic T lymphocytes; DCs: dendritic cells; mHSP70 407-426: microbial HSP70 peptide epitope 407–426; OK-432: a penicillin-inactivated streptococcus pyogenes; Th1: T helper 1; IL-2: interleukin-2; TEX: tumor-derived exosome; IFN-γ: interferon-gamma; IL-10: interleukin-10; TGF-β: transforming growth factor β; HMGN1: high-mobility group nucleosome-binding protein 1; DEX: DC-derived exosomes; P47-P: HCC-targeting peptide; AFP212-A2: α-fetoprotein epitope; N1ND-N: high-mobility group nucleosome-binding protein 1; TLR7: Toll-like receptor 7; TLR9: Toll-like receptor 9; pDCs: plasmacytoid dendritic cells; pDC: plasmacytoid dendritic cell; IFN-α: interferon alpha; NK cells: natural killer cells; PEI: percutaneous ethanol injection; RFTA: radiofrequency thermal ablation; IL-1β: interleukin-1beta; MDCs: myeloid dendritic cells; NF-κB: nuclear factor kappa-B; PD-1: programmed death-1; WNV: West Nile virus; HCC: hepatocellular carcinoma; CTL:cytotoxic T lymphocyte; DOX: doxorubicin; ICG: indocyanine green; BHM: bovine albumin/hyaluronan; ICD: immunogenic death; TME: tumor microenvironment; SSZ: sulfasalazine; CH-OD: chitosan hydrochloride and oxidized dextran; DAMPs: damage-associated molecular patterns; MOF-CpG-DMXAA: nanoparticles assembled from cytosine-phosphate-guanine oligodeoxyribonucleotides, 5,6-dimethylheteroanthrone-4-acetic acid, and metal–organic framework 801; TAMs, tumor-associated macrophages.

## 6. Conclusions and Future Perspectives

In this review, DCs activation and the influencing factors in HCC microenvironments were analyzed and summarized, and the activation strategies for DCs in the context of HCC immunotherapy were outlined, which are instrumental in guiding research on DC-based immunotherapies for HCC. Despite the notable achievements of DC vaccines in HCC immunotherapy, their high cost and lengthy preparation time pose challenges, increasing the risk of tumor immune escape and potentially leading to the proliferation of tumor immunosuppressive cells. Encouragingly, the emergence of exosomes and emerging nanocarriers brings hope for DC-based immunotherapy, facilitating easier antigen loading, effectively activating DCs, and eliciting robust and enduring tumor immunity, while also reversing the immunosuppressive tumor microenvironment. Furthermore, nanotechnology can be combined with traditional therapeutic modalities triggering immunogenicity or other immunotherapies to evoke potent anti-tumor immunity. In conclusion, the activation of DCs plays an important role in the induction of innate immunity and adaptive immunity against HCC. By optimizing DCs activation measures in HCC immunological microenvironments, novel breakthroughs in HCC immunotherapy are anticipated.
